# Diabetes Mellitus and Its Cardiovascular Complications: New Insights into an Old Disease

**DOI:** 10.1155/2019/1905194

**Published:** 2019-05-19

**Authors:** Celestino Sardu, Claudio De Lucia, Markus Wallner, Gaetano Santulli

**Affiliations:** ^1^University of Campania “Luigi Vanvitelli”, Naples, Italy; ^2^Center for Translational Medicine, Lewis Katz School of Medicine, Temple University, Philadelphia, PA, USA; ^3^Medical University of Graz, Graz, Austria; ^4^Albert Einstein College of Medicine, The “Norman Fleischer” Institute for Diabetes and Metabolism, The “Wilf Family” Cardiovascular Research Institute, New York, NY, USA; ^5^University of Naples “Federico II”, Naples, Italy

There are ~415 million people living with diabetes mellitus worldwide, with type 2 diabetes (T2DM) accounting for more than 90% of diabetic patients [1, 2]. T2DM negatively affects the prognosis of patients by markedly increasing both hospitalization and mortality rate [1]. The common phenotype of T2DM is characterized by relative insulin deficiency caused by pancreatic *β*-cell dysfunction and insulin resistance in target organs [2–4]. These aspects eventually cause an altered glucose homeostasis, with consequent systemic negative effects on molecular and cellular functions [5–7]. Coming in the merit of the present editorial, we edited the Special Issue “Diabetes Mellitus and Its Cardiovascular Complications: New Insights into an Old Disease,” collecting the state-of-the-art research in the field. Indeed, T2DM is a relevant cardiovascular (CV) risk that is known to be the leading cause of morbidity and mortality associated with T2DM [8]. Insulin resistance and hyperglycemia work together as continuous negative triggers impairing ionic channel activity, the epigenetic program, and the cellular function of several organs [8]. At the clinical level, T2DM is strongly associated with both micro- and macrovascular complications, including retinopathy, nephropathy, and neuropathy, as well as cerebrovascular disease, ischemic heart disease (IHD), and peripheral artery disease (PAD) [1, 9]. Several important concepts need to be highlighted: (1) diabetic nephropathy, cardiomyopathy, and PAD are frequently diagnosed at later disease stages; (2) screening programs are inconsistent and often inadequate to reduce the burden of these disorders [1]; (3) therefore, diet, exercise training, and lifestyle changes remain useful tools in preventing or at least delaying CV complications of T2DM [1, 10]. Remarkably, dysfunctional ionic channels can be detected in T2DM patients without structural heart disease by direct alterations of ionic currents [11] as well as in patients with concomitant heart failure (HF) [12]. T2DM might increase the risk of atherosclerosis [1] alongside with a loss of regenerative myocardial muscle functions during an acute coronary syndrome. Intriguingly, T2DM might also cause functional alterations in the absence of obstructive coronary stenosis [13]. Indeed, altered glucose homeostasis and insulin resistance might trigger an advanced atherosclerosis in coronary arteries in cases with obstructive coronary stenosis and also in patients with nonobstructive coronary stenosis [13, 14]. To date, T2DM has been shown to determine abnormalities in the dynamic responses to vasoactive stimuli, leading to increased rates of major adverse cardiac events (MACE) [13–15].On the other hand, T2DM might cause complex electrical alterations in HF patients increasing the risk of atrial and ventricular arrhythmias [16]. Specifically, T2DM induces alterations of ionic currents affecting action potential genesis and propagation in cardiac chambers, increasing automaticity and reentry mechanisms and favoring both atrial and ventricular arrhythmias [16]. Additionally, the increased inflammation and advanced cardiac fibrosis can lead to mechanical abnormalities of cardiac muscle with severe pump failure as well as higher rate of congestive HF and hospital admissions for HF worsening [15, 16]. In this setting, new drug therapies such as interventional treatments have been developed to revert these negative conditions in order to ameliorate not only coronary and cardiac function but also clinical prognosis in IHD and HF patients with T2DM [14–16]. Moreover, there is an increasing necessity to develop new diagnostic tools for early detection of CV complications as well as new efficient treatments for this pathological condition. Thereby, a better understanding of specific diabetes genotypes and phenotypes might result in more specific and tailored management of T2DM patients [1]. Hence, there is an urgent need to find new therapeutic approaches to blunt the systemic and tissue-specific effects of hyperglycemia and insulin resistance and to reduce the development of diabetic CV complications. In conclusion, we believe that the main goal in the near future will be to find treatments better tailored to diabetic patients using a personalized-medicine approach.
